# The flow of the Berry curvature vector field

**DOI:** 10.1038/s41598-021-04076-z

**Published:** 2022-01-07

**Authors:** Ondřej Stejskal, Martin Veis, Jaroslav Hamrle

**Affiliations:** Faculty of Mathematics and Physics, Charles University, Prague, Czech Republic

**Keywords:** Electronic properties and materials, Electronic structure, Ferromagnetism

## Abstract

The concept of Berry phase and Berry curvature has become ubiquitous in solid state physics as it relates to variety of phenomena, such as topological insulators, polarization, and various Hall effects. It is well known that large Berry curvatures arise from close proximity of hybridizing bands, however, the vectorial nature of the Berry curvature is not utilized in current research. On bulk bcc Fe, we demonstrate the flow of the Berry curvature vector field which features not only monopoles but also higher dimensional structures with its own topological features. They can provide a novel unique view on the electronic structure in all three dimensions. This knowledge is also used to quantify particular contributions to the intrinsic anomalous Hall effect in a simple analytical form.

## Introduction

The Berry phase has been discovered already in 1984^1^ but only in the last two decades, this concept found its way into the solid state physics. It ignited the search for topological properties of electrons in solids and resulted in several novel discoveries in various fields. It found success especially in lower dimensional systems and became the main focus in research areas such as topological insulators^2–5^, photonic crystals^6–11^, and Weyl semimetals^12–16^.

In three dimensions (3D), the *k*-space Berry connection of band *n* is defined by $\mathbf {A}^n(\mathbf {k})=i\langle {u_{n\mathbf {k}}} |\nabla _{\mathbf {k}}| {u_{n\mathbf {k}}} \rangle$, where $u_{n\mathbf {k}}$ are the cell-periodic Bloch functions. The gauge invariant Berry curvature is defined as the curl of the connection:

1 $$\begin{aligned} {\varvec{\Omega }}^n(\mathbf {k})=\nabla _{\mathbf {k}}\times \mathbf {A}^n(\mathbf {k}) \end{aligned}$$

and thus it is divergenceless except for monopoles. Its flux over any closed surface is quantized as invoked by the Chern theorem $\oint {\varvec{\Omega }}^n(\mathbf {k})\cdot \mathrm {d}\mathbf {S}=2\pi C_n$, where $C_n$ is an integer known as the Chern number or the Chern index and is a topological invariant^17^.

In bulk ferromagnetic metals, the Berry curvature acts as a magnetic field in parameter space^18,19^ resulting in the anomalous Hall effect (AHE) that is nowadays routinely formulated in Berry terms^20–23^. It is known that the Berry curvature is large wherever two bands approach each other energetically^20^. However, its full vectorial nature is not utilized in current research as the studies of the Berry curvature are limited to its component along the magnetization, $\Omega _z$.

In this theoretical work, we present ab-initio calculations of Berry curvature in bulk bcc Fe and demonstrate the benefits of its fully vectorial nature. We show higher dimensional structures of the Berry curvature vector field that are directly related to the electronic structure. This provides a novel and complementary view on the electronic structure in 3D and it can serve as a valuable alternative to the conventional 1D band structure plots. Furthermore, we demonstrate the application of the Berry curvature vector field in quantifying particular contributions to the intrinsic AHE.

**Figure 1:**
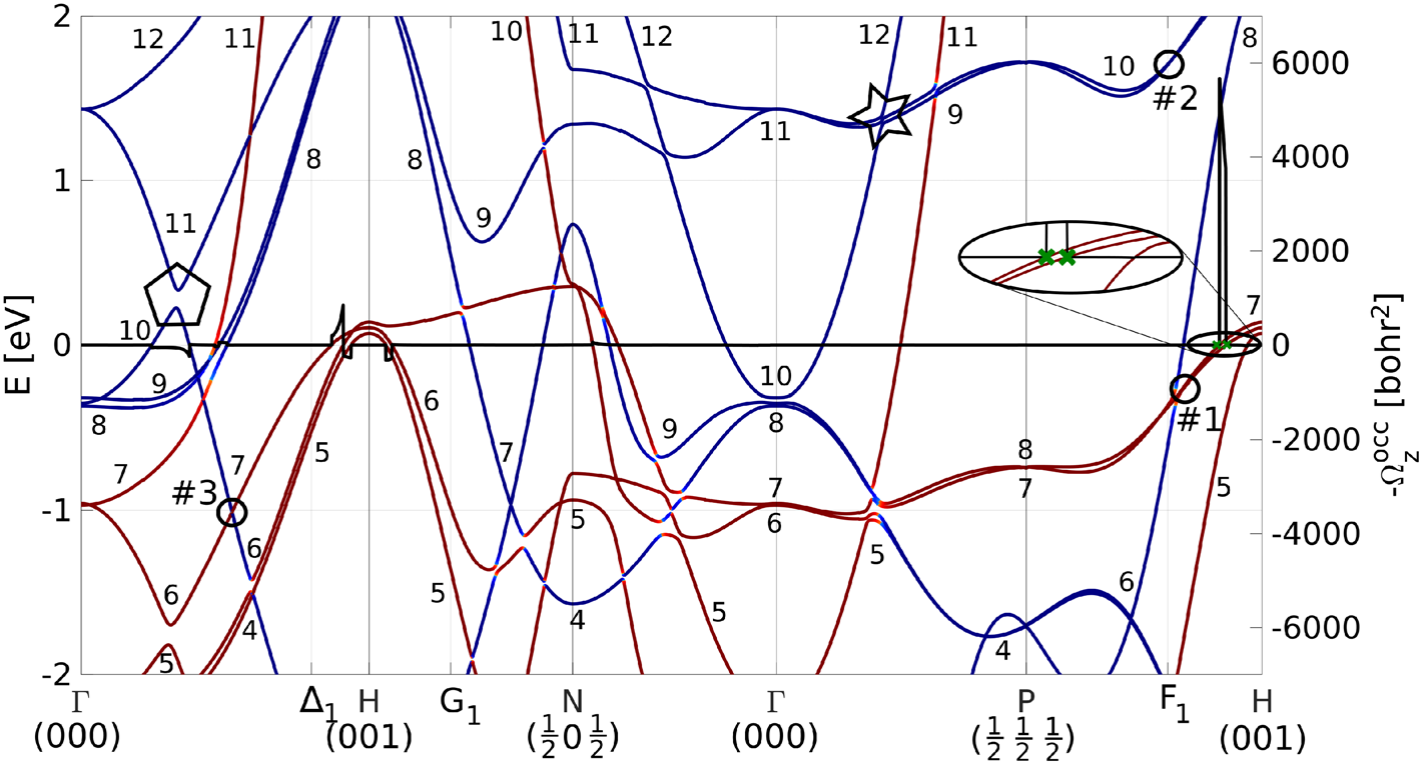
Conventional band structure of bcc Fe along a standard 1D-path in the Brillouin zone. Bands are indexed by their increasing energy value starting with 4*s* bands. Three additional *k*-points are marked on the horizontal axis, namely $\Delta _1=(0,0,0.8)$, $G_1=(0.2,0,0.8)$, and $F_1=(0.2,0.2,0.8)$. The red (blue) color corresponds to spin down (up). The black curve is the *z*-component of the Berry curvature over occupied states $\Omega _z^{\mathrm {occ}}=\Sigma _n f_n \Omega _z^n$. Selected Berry curvature monopoles are highlighted by circles and numbered. The investigated contribution to the intrinsic AHE is marked by black ellipse. The green crosses mark *k*-points where bands 6 and 7 cross the Fermi level (zoomed). Pentagon and star highlight studied features of the electronic structure. All labels are in conjunction with Figs. 2, 3.

## Dimensionality of the Berry curvature vector flow

The numerical calculations are performed on bulk bcc Fe with magnetization in the *z*-direction (details in Methods). Conventional band structure is shown in Fig. 1 along the standard *k*-path in the Brillouin zone.

**Figure 2:**
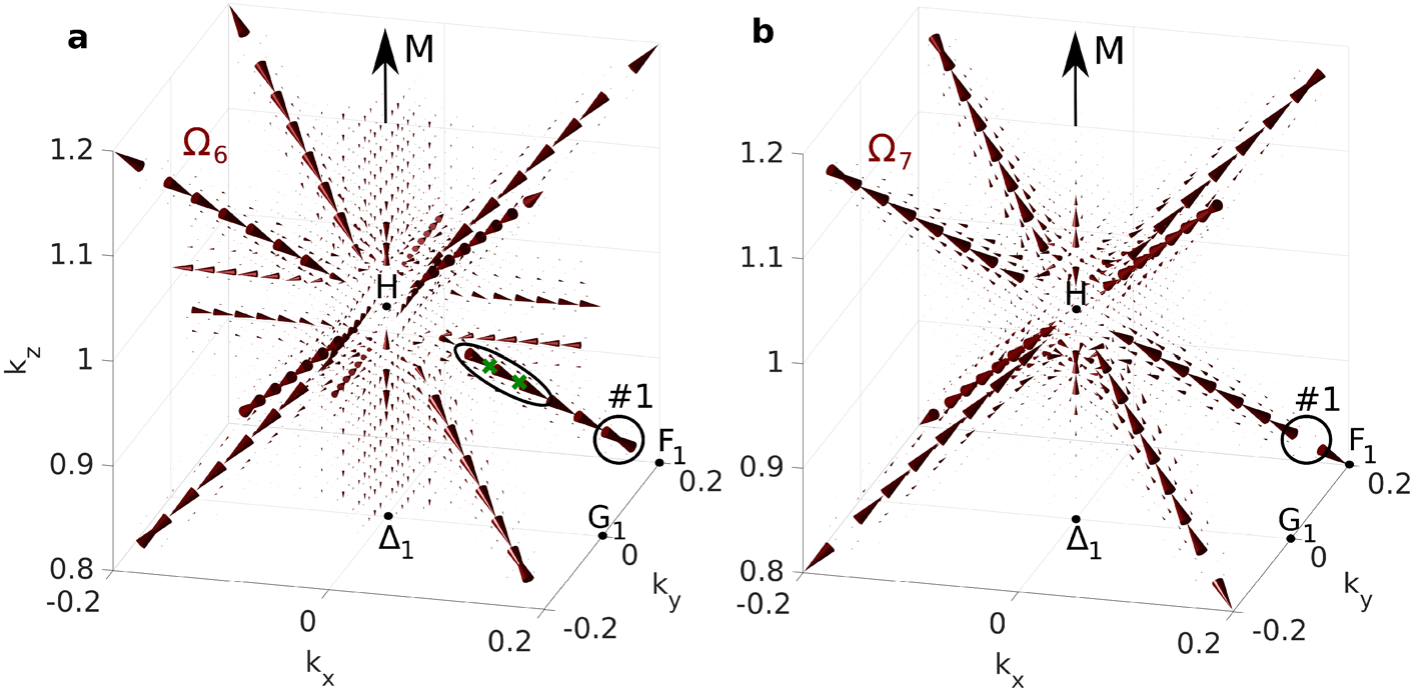
Flow of the Berry curvature vector field. Berry curvature of (**a**), band 6 and (**b**), band 7 in a cube centered at point H. All relevant features are highlighted and relate to Fig. 1. The size of the largest cone is 3500 $\hbox {bohr}^2$. Cone size cutoff was performed as in the vicinity of monopole sources the magnitude of the Berry curvature is very large.

The calculated Berry curvature vector fields are depicted in Fig. 2. The Berry curvature is represented by cones pointing in the direction of the (pseudo)vector $(\Omega _x,\Omega _y,\Omega _z)$ with size proportional to its magnitude. In (a), the Berry curvature of band 6 is shown in the vicinity of point H, in (b) the Berry curvature of band 7 in the same volume. We have identified three types of Berry curvature flow based on its dimension denoted (0D, 1D, 2D).

*1D flow*: The most dominant flows in Fig. 2 are the 1D flows that appear along high symmetry lines, namely $\Gamma$-*H*, $\Gamma$-*P* and *H*-*P*, where bands are brought together by symmetry and split by the spin-orbit interaction. Several 1D Berry curvature flows of bands 6 and 7 are shown in Fig. 2a,b respectively. The most localized and thus strongest 1D flow is in the H-P (i.e. H-$\hbox {F}_1$) direction. It is generated by hybridization of bands 6 and 7, see the band structure in Fig. 1. The flux is confined to the line and its immediate vicinity and is tangent to the line, i.e., to the manifold of maximal band hybridization. This flow also contributes to the intrinsic AHE which will be discussed below. Note that for the adjacent band 7, the Berry curvature flows in the opposite direction (Fig. 2b). Another 1D flow appears along $\Gamma$-H line parallel to the magnetization $\mathbf {M}$; this flow is less localized due to simultaneous hybridization of three bands 5, 6 and 7. Another type of 1D flow appears on the $\Gamma$-H line perpendicular to the magnetization that exhibits as two flows in the opposite direction. This situation arises when the bands are degenerate along the line.

1D flow is analytically described by two-level Hamiltonian $H=\sum _i H_i \sigma _i$, $i=\{1,2,3\}$ where $\sigma _i$ are Pauli matrices. The eigenenergies are $\epsilon _\pm =\pm \sqrt{H_1^2+H_2^2+H_3^2}$. We assume two hybridizing bands split by the spin-orbit interaction along $k_z$ while diverging linearly in $k_x$ and $k_y$ directions: $H_1=Qk_x$, $H_2=Qk_y$, $H_3=S$ (i.e. with $S=0$, the bands would touch along $k_z$). Then, the Berry curvature has only *z*-component and its profile is given by:

2 $$\begin{aligned} \Omega _z^\pm (k_x,k_y)=\frac{SQ^2}{2\epsilon _\pm ^{3}}=\pm \frac{SQ^2}{2(Q^2k_x^2+Q^2k_y^2+S^2)^{3/2}}. \end{aligned}$$

The total flux of the flow is quantized:

3 $$\begin{aligned} \Phi =\int \mathrm {d}k_x\mathrm {d}k_y \Omega _z = \pi \,{{\mathrm {sgn}}}(S), \end{aligned}$$

where ${\mathrm {sgn}}(S)={-1,0,1}$ for $S<0$, $S=0$ and $S>0$, respectively. The total flux can acquire only quantized values of $\pm \pi$ or 0 and thus it is a topological invariant. Furthermore, this quantization is present for any 1D flux provided that the bands diverge energetically away from the hybridization axis.

**Figure 3:**
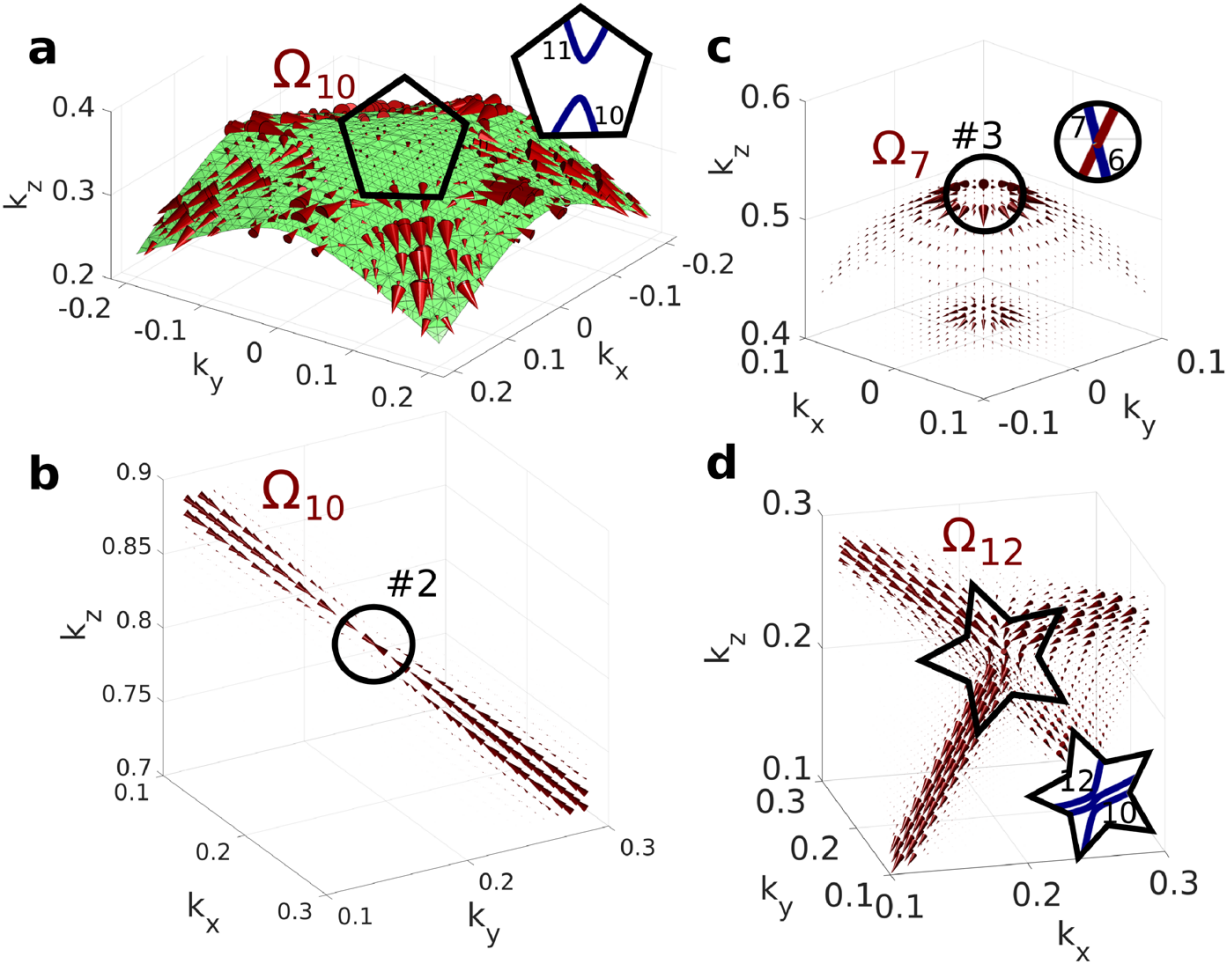
(**a**), Demonstration of the 2D flow of the Berry curvature of band 10 along a surface of maximal hybridization. (**b**), Berry curvature of band 10 exhibiting monopole drained via 1D flow. (**c**), Berry curvature of band 7 exhibiting monopole drained via 2D surface flow. (**d**), Berry curvature of band 12 demonstrating the transition of 2D flow into 1D flow. The respective band structure cutouts are shown in the insets. The size of the largest cone is 500 $\hbox {bohr}^2$ in (**a**), 1700 $\hbox {bohr}^2$ in (**b**), 2000 $\hbox {bohr}^2$ in (**c**), and 2600 $\hbox {bohr}^2$ in (**d**).

*2D flow*: Second type is the 2D flow where the Berry curvature flows tangentially to a surface. This is the most common type and it appears whenever band crossing is avoided by the spin-orbit interaction. Example is shown in Fig. 3a, where the Berry curvature of band 10 is depicted. It is generated by avoided crossing of bands 10 and 11 marked by black pentagon in Fig. 1. The flow is presented along with the guiding surface which is the surface of maximal hybridization of the respective bands and is defined as a set of *k*-points where these bands are maximally hybridized. Each band has a dominant character in the real orbital basis when isolated. If another band approaches energetically, these bands hybridize and their characters mix. At the surface of maximal hybridization the mixing is maximal, i.e. the real orbital basis coefficients corresponding to each of the band (if it was isolated) equal. It turns out that the Berry curvature is tangent to this surface. The 2D flow is generally weaker as the entire flux is now spread over a surface.

*0D flow*: Last, the well-known Berry curvature flow originated in monopoles (Weyl points) is discussed. These monopoles arise at points of band degeneracies and according to the Chern theorem, the outcoming flux is quantized and equal to $\pm 2\pi$ providing source and sink of the Berry curvature (there are also monopoles with $4\pi$ flux but they are rare in bcc Fe). The monopoles were found to be common in bcc Fe ^24^. For illustrative purposes, we show the Berry curvature flow from three selected monopoles: #1 originating in degeneracy of bands 6 and 7 on the H-P line; #2 that arises due to degeneracy of bands 9 and 10 on the H-P line; and #3 from degeneracy of bands 6 and 7 approximately in the middle of the $\Gamma$-H line. Note that each degeneracy provides two monopoles, one in each degenerating band, and thus sink in one band (e.g. #1 in Fig. 2a) is always accompanied by source in the other band (#1 in Fig. 2b).

## Relation to band structure

The Berry curvature is large wherever two or more bands approach energetically and hybridize^20^. However, the structure of the Berry curvature vector field carries additional information that is closely related to the band structure. Namely, based on our observations, the flow of the Berry curvature is tangent to the manifold of maximal hybridization (line in the 1D case and surface in the 2D case). Wherever the Berry curvature is close to zero, the studied band is energetically distant from other bands. If the band is approached by another band, the Berry curvature gets large and its flow reveals if the band is approached along a line or a surface or whether a monopole is generated by a point degeneracy. This information can be utilized in reading of the electronic structure of a particular band in all three dimensions and is not limited to a 1D path throughout the Brillouin zone, as it is done in conventional band structure plots (Fig. 1).

As examples, in Fig. 3 several scenarios demonstrating the relation between the band structure and the Berry curvature flow are shown. Fig. 3b demonstrates the Berry curvature originating in a monopole #2 and being drained away by a 1D flow. This situation arises when two bands are brought together by symmetry along higher symmetry line and split by the spin-orbit interaction anywhere but in the monopole. In Fig. 3c, the Berry curvature originating in a monopole #3 is drained away via a 2D flow, which indicates a pair of bands approached along a surface and split anywhere on the surface but in the monopole where the degeneracy occurs. Fig. 3d shows the transition from 2D to 1D flow. This occurs when a pair of bands is brought together by symmetry along a higher symmetry line (generating 1D flow) and is crossed by a third band (generating 2D flow) as highlighted by black star in Fig. 1.

The Berry curvature introduced so far is defined for non-degenerate bands^25^. Therefore, it is required that either time-reversal or inversion symmetry is broken. In materials with simultaneous inversion and time-reversal symmetry, there exist two orthogonal states (spin-up and spin-down) with the same energy at each *k*-point which is known as the Kramers degeneracy. However, it has already been demonstrated that if the non-Abelian Berry curvature is used in its tensor form, its determinant has analogical properties to the magnitude of the ordinary Berry curvature vector^25–27^, i.e. it gets larger wherever one Kramers doublet is energetically approached by another doublet. Establishing a quantity analogical to the direction of the vector enables this alternative visualisation of the electronic structure for any type of crystal.

## 1D flow contribution to anomalous Hall effect

Calculation of the anomalous Hall conductivity by integration of the Berry curvature over the Brillouin zone (BZ) $\sigma ^{\mathrm {AHE}}_{xy}=-\frac{e^2}{\hbar }\frac{1}{(2\pi )^3}\int _\mathrm {BZ} \Omega _z^\mathrm {occ}\,\mathrm {d}\mathbf {k}$ is nowadays a well-established procedure for evaluating the intrinsic part of the AHE^20–23^. AHE arises wherever the Fermi level lies inside the spin-orbit induced gap of the split bands (see $\Omega _z^\mathrm {occ}$ denoted by black line in Fig. 1). The quantized nature of the 1D flow of the Berry curvature field enables simple analytical expression for its contribution to AHE.

The studied 1D flow contribution comes from bands 6 and 7 along H-P line and is highlighted by black ellipse in Figs. 1, 2a. Both bands strongly hybridize along the line and induce very localized 1D Berry curvature flow of flux $\pi$. The Berry curvatures of bands 6 and 7 flow in the opposite directions (see Fig. 2), therefore when both bands lie below Fermi level, their Berry curvatures cancel and $\Omega _z^\mathrm {occ}=0$. Similarly, they do not contribute to AHE, if both bands are above Fermi energy. Thus, only when the Fermi level lies in the spin-orbit gap (denoted by green crosses), contribution to AHE is induced. The strong localization of the 1D flow makes the Berry curvature effectively a 1D feature allowing to express its contribution to AHE in a simple form:

4 $$\begin{aligned} \sigma ^\mathrm {AHE,1D}_{xy}=-\frac{e^2}{\hbar }\frac{1}{(2\pi )^3}\pi \Delta k \cos {\varphi },\quad \cos {\varphi }=\hat{\Omega }\cdot \hat{z}. \end{aligned}$$

This expression highlights the topological nature of the AHE in 3D materials via the quantized $\pi$ flux. $\Delta k$ is the reciprocal distance between the green crosses (i.e. where the lower band is occupied and simultaneously the upper band is unoccupied) and the $\cos {\varphi }$ term extracts the *z*-component from the direction of the flow. In the depicted case $\Delta k=0.0281$ $\hbox {bohr}^{-1}$ and $\cos {\varphi }=-1/\sqrt{3}$, as the Berry curvature flows in the $[11\overline{1}]$ direction. This provides AHE contribution from single 1D flow $\sigma ^\mathrm {AHE,1D}_{xy}=9.4304$ ($\Omega \hbox {cm}$)$^{-1}$. Due to symmetry, this contribution appears eight times in the BZ with the total strength of 75.4432 ($\Omega \hbox {cm}$)$^{-1}$ describing 10% of the total AHE (751 ($\Omega \hbox {cm}$)$^{-1}$ for bcc Fe^20^). This type of 1D contribution to AHE is common among crystals as is dictated by symmetry and it can be utilized in the band structure engineering. Note, that there are other contributions to AHE both 1D and 2D, however, these are specific to bcc Fe. The structure of the flows gets complicated and hence the presented analytical formula, Eq. (4), does not apply in this simple form.

## Conclusion

The flow of the Berry curvature vector field carries important information and should not be overlooked. We identified three types of Berry curvature flow based on its dimension as monopoles (0D), flows along a line (1D) which are quantized by $\pi$, and flows along a surface (2D). We demonstrated its use in two instances. First, as a complete view of the band structure in three dimensions that serves as complementary alternative to the conventional one-dimensional visualisations along particular *k*-path in the Brillouin zone. Second, part of AHE originates from 1D flow of the Berry curvature and can be described by simple expression highlighting its topological origin. While this formalism is currently applicable only to crystals with broken time-reversal or inversion symmetry, the generalization to non-Abelian Berry curvature has the potential to extend its applicability to all types of crystals.

## Methods

The electronic structure of bcc Fe is calculated by the WIEN2k code^28,29^ which uses the density functional theory and is based on the full-potential linearized augmented plane wave method. The only input into the calculation is the bcc Fe lattice constant of 2.8665 Å^30^. The electronic structure is calculated with 729,000 *k*-points in the full Brillouin zone and the local spin density approximation^31^ is used as the exchange-correlation potential. The calculation is performed with the spin-orbit interaction and with magnetization in the *z*-direction. The product of the smallest atomic sphere and the largest reciprocal space vector was set to $R_\mathrm {MT}K_\mathrm {max}=8$ with the maximum value of the partial waves inside the spheres $l_\mathrm {max}=10$. States up to 3*s* are treated as core states. Bands are labeled by an increasing energy eigenvalue starting with 4*s* states.

The Berry curvature is calculated by:

5 $$\begin{aligned} \Omega _{\mu \nu }^n(\mathbf {k})=-\frac{\hbar ^2}{m^2}\sum _{n'\ne n} \frac{2 \mathrm {Im}\langle {\psi _{n\mathbf {k}}} |p_{\mu }| {\psi _{n'\mathbf {k}}} \rangle \langle {\psi _{n'\mathbf {k}}} |p_{\nu }| {\psi _{n\mathbf {k}}} \rangle }{(E_n-E_{n'})^2}, \end{aligned}$$

where $E_n$ is the energy, $\psi _{n\mathbf {k}}$ are Bloch wave functions, and $p_\mu$ are momentum operators. The Berry curvature $\Omega _{\mu \nu }$, which is a field tensor, is in three dimensions related to its vector form via $\Omega _{\mu \nu }=\epsilon _{\mu \nu \xi }\Omega _\xi$. The bcc Fe crystal has inversion symmetry, thus the Berry curvature obeys ${\varvec{\Omega }}^n(\mathbf {k})={\varvec{\Omega }}^n(-\mathbf {k})$. However, the time-reversal symmetry is broken by the magnetization inducing non-zero Berry curvature in the Brillouin zone. With magnetization in the *z*-direction, *x* and *y* components of the Berry curvature vector vanish upon integration over the Brillouin zone and solely the *z*-component gives rise to the anomalous Hall conductivity.
