# Publisher Correction: The flow of the Berry curvature vector field

**DOI:** 10.1038/s41598-022-15355-8

**Published:** 2022-06-29

**Authors:** Ondřej Stejskal, Martin Veis, Jaroslav Hamrle

**Affiliations:** Faculty of Mathematics and Physics, Charles University, Prague, Czech Republic

Correction to: *Scientific Reports* https://doi.org/10.1038/s41598-021-04076-z, published online 07 January 2022

The original version of this Article contained errors in the Reference list, where the titles of references 1,6,9,11,18,19,20,21, and 25 were incorrectly given as

1. Berry, M. V. Proceedings of the royal society of London. *A. Math. Phys. Sci.* **392**, 45 (1984).

6. Wang, Z., Chong, Y., Joannopoulos, J. D. & Soljačić, M. RNA-Seq: A revolutionary tool for transcriptomics. *Nature* **461**, 772 (2009).

9. Wimmer, M., Price, H. M., Carusotto, I. & Peschel, U. Engineering hybrid epitaxial InAsSb/Al nanowires for stronger topological protection. *Nature* **13**, 545 (2017).

11. Rong, K. *et al.* Probing nanoscale fluctuation of ferromagnetic meta-atoms with a stochastic photonic spin Hall effect. *Nat. Nanotechnol.* **15**, 927 (2020).

18. Bliokh, K. & Bliokh, Y. Theory of superconductivity. A treatise on the magnetic vector potential. *Ann. Phys.* **319**, 13 (2005).

19. Price, H. M., Ozawa, T. & Carusotto, I. Topological photonics. *Phys. Rev. Lett.* **113**, 190403 (2014).

20. Yao, Y. *et al.* Non-collinear antiferromagnets and the anomalous Hall effect. *Phys. Rev. Lett.* **92**, 037204 (2004).

21. Nagaosa, N., Sinova, J., Onoda, S., MacDonald, A. H. & Ong, N. P. Non-collinear antiferromagnets and the anomalous Hall effect. *Rev. Mod. Phys.* **82**, 1539 (2010).

25. Gradhand, M. *et al.* Non-collinear antiferromagnets and the anomalous Hall effect. *J. Phys. Condens. Matter* **24**, 213202 (2012).

The correct references now read:

1. Berry, M. V. Quantal phase factors accompanying adiabatic changes. *Proc. R. Soc. Lond. A. Math. Phys. Sci*. **392**, 45–57 (1984).

6. Wang, Z., Chong, Y., Joannopoulos, J. D. & Soljačić, M. Observation of unidirectional backscattering-immune topological electromagnetic states. *Nature* **461**, 772 (2009).

9. Wimmer, M., Price, H. M., Carusotto, I. & Peschel, U. Experimental measurement of the Berry curvature from anomalous transport. *Nature* **13**, 545 (2017).

11. Rong, K. *et al.* Photonic Rashba effect from quantum emitters mediated by a Berry-phase defective photonic crystal. *Nat. Nanotechnol. 15*, 927 (2020).

18. Bliokh, K. & Bliokh, Y. Spin gauge fields: From Berry phase to topological spin transport and Hall effects. *Ann. Phys.* **319,** 13-47 (2005).

19. Price, H. M., Ozawa, T. & Carusotto, I. Quantum mechanics with a momentum-space artificial magnetic field. *Phys. Rev. Lett.* **113**, 190403 (2014).

20. Yao, Y. *et al.* First principles calculation of anomalous Hall conductivity in ferromagnetic bcc Fe. *Phys. Rev. Lett.* **92**, 037204 (2004).

21. Nagaosa, N., Sinova, J., Onoda, S., MacDonald, A. H. & Ong, N. P. Anomalous Hall effect. *Rev. Mod. Phys.* **82**, 1539-1592 (2010).

25. Gradhand, M. *et al.* First-principle calculations of the Berry curvature of Bloch states for charge and spin transport of electrons. *J. Phys. Condens. Matter* **24**, 213202 (2012).

The original Article has been corrected.

